# The A to I editing landscape in melanoma and its relation to clinical outcome

**DOI:** 10.1080/15476286.2022.2110390

**Published:** 2022-08-21

**Authors:** Austeja Amweg, Marina Tusup, Phil Cheng, Ernesto Picardi, Reinhard Dummer, Mitchell P Levesque, Lars E French, Emmanuella Guenova, Severin Läuchli, Thomas Kundig, Mark Mellett, Steve Pascolo

**Affiliations:** a Department of Dermatology, University Hospital Zürich (USZ), Zürich, Switzerland; b Faculty of Medicine, University of Zürich (UZH), Zürich, Switzerland; c Department of Biosciences, Biotechnology and Biopharmaceutics, University of Bari “A. Moro”, Bari, Italy; d Institute of Biomembranes, Bioenergetics and Molecular Biotechnologies (IBIOM), National Research Council, Bari, Italy; e Department of Dermatology and Allergy, University Hospital, LMU Munich, Munich, Germany; f Dr. Philip Frost, Department of Dermatology and Cutaneous Surgery, University of Miami Miller School of Medicine, Miami, FL, USA; g Department of Dermatology, Lausanne University Hospital (CHUV) and University of Lausanne, Lausanne, Switzerland

**Keywords:** ADAR, A-to-I editing, Alu sequences, Editing, melanoma, immune checkpoint inhibitors, immunotherapy

## Abstract

RNA editing refers to non-transient RNA modifications that occur after transcription and prior to translation by the ribosomes. RNA editing is more widespread in cancer cells than in non-transformed cells and is associated with tumorigenesis of various cancer tissues. However, RNA editing can also generate neo-antigens that expose tumour cells to host immunosurveillance. Global RNA editing in melanoma and its relevance to clinical outcome currently remain poorly characterized. The present study compared RNA editing as well as gene expression in tumour cell lines from melanoma patients of short or long metastasis-free survival, patients relapsing or not after immuno- and targeted therapy and tumours harbouring *BRAF* or *NRAS* mutations. Overall, our results showed that *NTRK* gene expression can be a marker of resistance to BRAF and MEK inhibition and gives some insights of candidate genes as potential biomarkers. In addition, this study revealed an increase in Adenosine-to-Inosine editing in Alu regions and in non-repetitive regions, including the hyperediting of the *MOK* and *DZIP3* genes in relapsed tumour samples during targeted therapy and of the *ZBTB11* gene in NRAS mutated melanoma cells. Therefore, RNA editing could be a promising tool for identifying predictive markers, tumour neoantigens and targetable pathways that could help in preventing relapses during immuno- or targeted therapies.

## Introduction

RNA editing is a molecular process through which cells can make discrete changes to specific nucleotide sequences within RNA molecules after they have been generated by RNA polymerase. It includes nucleobase modifications such as cytidine (C) to uridine (U) and adenosine (A) to inosine (I) deaminations, as well as non-template nucleotide additions and insertions. Editing in messenger RNA (mRNA) permits the development of cell- or context-specific alterations in protein sequences or expression levels without requiring underlying genomic DNA changes. Editing in mRNA contributes to important physiological processes increasing transcript and protein diversification but also by eventually fuelling the progression of tumour cells [1–4].

Deamination of RNA converts cytosine (C) to uracil (U) and adenine (A) to inosine (I), that can lead to missense and nonsense mutations as well as to changes in the structure of the mRNA and its fate [5,6]. C-to-U editing is catalysed by the activation induced cytidine deaminase/apolipoprotein B editing complex (AID/APOBEC) family [7,8] and only a few instances have been identified in the human transcriptome. A-to-I modifications, instead, are frequent. They are performed by adenosine deaminases acting on RNA (ADAR) enzymes that catalyse adenosine hydrolytic deamination in double-stranded nucleic acid structures. The ADAR family consists of three members, ADAR1, of which there are two isoforms, ADAR1p150 and ADAR1p110, ADAR2 and ADAR3. All three ADAR proteins have deaminase domains but only ADAR1 and ADAR2 are reported to induce A-to-I editing in mammals [9–11]. ADAR1 and ADAR2 are encoded by the *ADAR* and *ADARB1* genes, repectively. While ADAR1 is ubiquiteously expressed, ADAR2 is mostly expressed in cells of the central nervous system (CNS) [10,12]. Adenosine deaminases that act on tRNAs (ADATs) form another class of A-to-I enzymes, which specifically target transfer RNA [13].

A-to-I RNA editing is essential for the maintenance of cellular homoeostasis and processes, including brain development and embryonic erythropoiesis [14–16]. Indeed, *Adar1*-deficient mice are embryonically lethal [14,17]. In humans A-to-I RNA editing is prominent and the majority of events occur in the primate-specific *Arthrobacter luteus* (Alu) sequences. Indeed, A-to-I conversion in Alu regions is the most common type of RNA editing accounting for ~97% of global editing events [18]. Alu sequences are typically 300 bp long and are the dominant form of short interspersed nuclear elements (SINEs) in primates [19]. At a million copies, Alu sequences can comprise 10% of the human genome and they are more abundant in gene-rich regions [20]. Pairing of oppositely orientated Alu sequences can produce RNA duplexes, which are ideal targets for ADAR enzymes. ADAR editing is common in introns and the 3’ and 5’ untranslated regions (UTRs) of mRNAs. These non-coding editing events are important and can affect mRNA stability, splicing, gene expression or recognition by microRNAs (miRNAs) [21].

Additionally, A-to-I editing by ADARs has been shown to occur in non-repetitive sequences, leading to at least 50 different recoding events known in human cells [22,23]. For example, ADAR1 alters the amino acid sequence of the DNA repair enzyme, Nei like DNA glycosylase 1 (NEIL1) and ADAR2 edits the mRNA coding the glutamate ionotropic Receptor AMPA type subunit 2 (Gria2) *(mostly at the Q/R site – position 607*), this conversion makes the AMPAR Ca^2+^-impermeable, which has a huge impact on the function [15,24].

Several differential editing events have been reported in tumorigenesis, such as *antizyme inhibitor 1* (*AZIN1)* editing in liver cancer [1], *cell division cycle 14B (CDC14B)* editing in glioblastoma [25], *RAS homolog family member Q (RHOQ)* editing in colorectal cancer [26], *gamma-aminobutyric acid type A receptor subunit aplha3 (GABRA3)* editing in breast cancer [27], *solute carrier family 22 member 3 (SLC22A3)* and *insulin-like growth factor binding protein 7* (*IGFBP7)* editing in oesophageal cancer [28] and *podocalyxin-like 1* (*PODXL)* editing in gastric cancer [29]. Such abberations in RNA editing may have advantages over DNA mutations by bestowing cancer cells with dynamic plasticity, beneficial at certain stages of growth and changing microenvironments.

It is known that ADAR1 can have a dual effect on cancer progression by participating both in immunosuppression or by facilitating neo-antigen formation [30–33]. Editing of double-stranded RNA by ADAR1 prevents stimulation of innate immune pattern recognition receptors such as the Retinoic acid-inducible gene I (RIG-I) family, favouring immune-silencing [34]. Meanwhile, for melanoma, ADAR1 is known to act as a tumour suppressor by targeting miR455-5p and miR378a-3p that contribute to melanoma progression [35,36]. Indeed, a downregulation of ADAR1 is typically observed in melanoma [2,30,37]. Moreover, Zhang and colleagues demonstrated that ADAR1-mediated over-editing of the mRNA coding cyclin I could generate peculiar MHC-presented epitopes in melanoma cells for detection by the immune system [31]. These findings suggest that expression of ADAR1 in metastatic melanoma cells could exert opposing roles for metastatic growth.

Immunotherapies and targeted therapies have been validated for the treatment of metastatic melanoma. Immune checkpoint blockade therapy includes antibodies that block cytotoxic T-lymphocyte-associated protein 4 (CTLA-4), programmed cell death-1 receptor (PD-1), programmed cell death-ligand1 (PD-L1) and are widely used in melanoma treatment. Ipilimumab binds to CTLA-4 on T cells, which enhances the anti-tumour immune response, whereas monoclonal antibodies such as Pembrolizumab and Nivolumab exert anti-tumour immune response by blocking PD-1 and PD-L1 signalling [38]. Targeted therapies specifically inhibit the driver mutations of carcinogenesis. Vemurafenib is an agent approved for the BRAF V600E activating mutation and inhibits the kinase activity that is responsible for hyperactivating the mitogen-activated protein kinase (MAPK) pathway. Its use in combination with Cobimetinib, a mitogen-activated protein kinase kinase (MEK) pathway inhibitor, has been associated with improvement of progression-free survival, in comparison to Vemurafenib monotherapy [39]. Combination therapies, such as Encorafenib (LGX818) with Binimetinib (MEK162), demonstrate long-term efficacy in patients with advanced BRAF V600 mutated melanoma, by blocking the mutated BRAF kinase protein and MEK1/2, respectively [40]. MEK162 is also evaluated in combination with third generation cyclin dependent kinases 4 and 6 (CDK4/6) inhibitor, Ribociclib (LEE001), showing increased antitumour activity and safety in advanced *NRAS* and *BRAF* mutated melanoma [41].

Although immunotherapy and targeted therapies are associated with high response rates in metastatic melanoma therapy, most responses are not durable [42]. The shortfall of long-term therapeutic efficacy is attributed to manifestation of resistance, acquired *via* genetic and epigenetic mechanisms [43,44]. Evidence also points to epitranscriptomic mechanisms that permit the adaptation of the tumour cells [33]. Previously, we reported that in melanoma, Alu editing correlates with relapse during immune checkpoint inhibitor treatment [45]. Interestingly, ADAR2 expression was increased in these relapsed tumours.

To date, RNA editing events in melanoma related to clinical outcome are still largely underinvestigated. Therefore, we sought to study RNA editing of melanoma cell lines derived from patients based on clinical survival, response to treatment and mutation status.

## Results

### Clinical data

To assess differential gene expression and editing events in melanoma, biopsies were collected from 67 progressing stage III–IV melanoma patients and were used to generate cell lines. Among patients, 31 (46,3%) were males and 36 (53,7%) were females, the median age was 56 (±16) years (Table 1). All biopsies analysed were from metastatic lesions. Melanomas were characterized for the genetic status (WT versus mutated) of BRAF, NRAS, cKIT as well as GNAQ and GNA11 based on the previously described method [46]. Patients were either untreated (n = 42) or received immune checkpoint inhibitors (n = 15), targeted therapy (n = 9), which included Vemurafenib (BRAF V600E inhibitor) used in combination with Cobimetinib (MEK inhibitor(i), Encorafenib (LGX818, BRAFi) with Binimetinib (MEK162, MEKi) and Ribociclib (LEE001, CDK4/6i) or Encorafenib (LGX818, BRAFi) with only Binimetinib (MEK162, MEKi) or a combination of both (n = 1) in the 6 months prior to biopsy. Overall time of survival (from first melanoma diagnosis) among patients who have died from their cancer was as follows: 20% lived less than 2 years, 34.5% lived between 2 to 5 years and 45.5% lived over 5 years. The 67 generated cell lines were characterized for homogeneity as previously described [45]. PolyA+ RNAs were sequenced by next generation sequencing (RNAseq) and sequence data were used for gene expression and editing analysis.

**Table 1.** Demographics and characteristics of melanomas used in this study.

	Group	N	%
Gender	Male	31	46,26
	Female	36	53,73
	Total	67	100
Mutation	BRAF	40	59,70
	NRAS	16	23,88
	cKIT	4	5,97
	WT	4	5,97
	Double	1	1,49
	GNAQ	1	1,49
	GNA11	1	1,49
	Total	67	100
Treatment	Immunotherapy	15	22,38
	Targeted Therapy	9	13,43
	Mixed Therapy	1	1,49
	No Therapy	42	62,68
	Total	67	100
Survival	Living	12	17,91
	<2 years	11	16,41
	2–5 years	19	28,35
	>5 years	25	37,31
	Total	67	100

Patients and corresponding cell lines. The table presents the gender, Mutation status, Treatment and Survival of patients used in this study from whom the cell lines were isolated.

### Differential gene expression

Three main molecular pathways are habitually dysregulated in melanoma. These include the RAS-RAF-MEK-ERK pathway (invariably through mutation of *BRAF, NRAS* or *receptor tyrosine kinase* (*cKIT)*), the cyclin-dependent kinase (CDK) inhibitor 2A (CDKN2A)-CDK4-retinoblastoma (RB) pathway (through mutation of *CDKN2* or *CDK4*), and the alternative reading frame (ARF) -p53 pathway (due to mutations in the corresponding *ARF* or *TP53* genes).

Other pathways such as the phosphoinositide 3-kinase (PI3K)-AKT pathway (through mutation of *NRAS, phosphatase and tensin homolog* (*PTEN*) or *PIK3 catalytic subunit alpha* (*PI3CA*)) and the canonical Wnt signalling pathway (due to mutation of *catenin beta 1* (*CTNNB1)* or *adenomatous polyposis coli* (*APC)* genes) have been also associated with melanocytic tumours to a lesser degree [47]. It was of interest to determine in our dataset, which pathways would be affected based on mutation status, treatment or survival.

Therefore, the 67 RNAseq datasets were grouped according to mutation status, treatment or survival and studied using multivariant analysis of limma and voom. Based on q-value <0.05 and p-value <0.001, no differentially expressed genes (DEGs) were found to be significantly up- or down-regulated between NRAS-mutated, BRAF-mutated cell lines and lines derived from melanomas harbouring other mutations (cKIT, G protein subunit alpha q (GNAQ) or G protein subunit alpha 11 (GNA11) or no mutations (wild-type), grouped together here as ‘Other’ (Supplementary Figure 1 shows results for q value >0.05, thus considered not significant).

**Figure 1.**
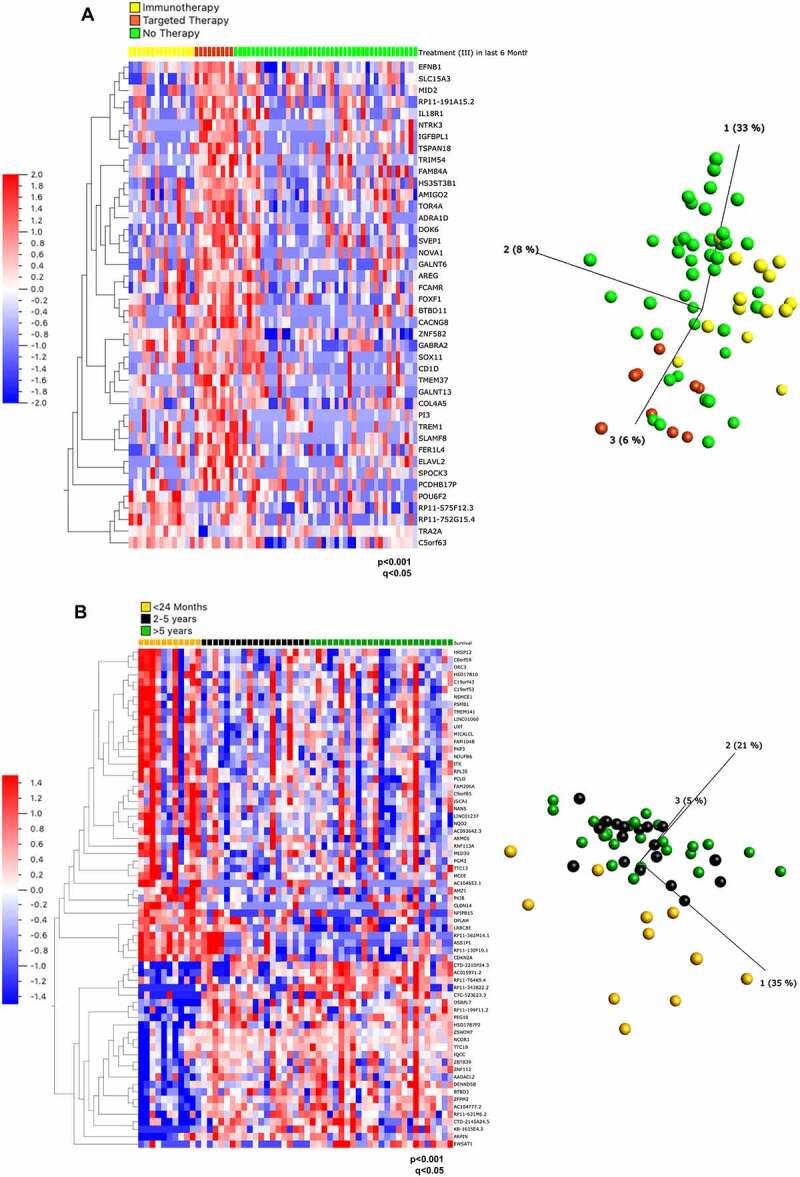
Gene expression analysis of cell lines from patients based on mutation status, treatment and survival. (**A**) Heat map (left) and PCA plot (right) representation of gene expression in cell lines from 66 patients divided into 3 treatment groups: relapsing after immunotherapy, after targeted therapy or prior to any therapy ‘no therapy’ showed 42 differentially expressed genes ranked based on q values obtained through multivariant analysis limma and voom. (**B**) Heat map (left) and PCA plot (right) representation of gene expression in cell lines from 55 patients divided into 3 survival groups showed 77 differentially expressed genes ranked based on q values obtained through multivariant analysis limma and voom. The gene *FAM84A* is now known as *LRATD1, AC104653*.1 as *ACTR3-AS1, C19orf43* as *TRIR, HRSP12* as *RIDA, MICALCL* as *MICAL2*, C*TD-2210P24*.3 as *LINC02690* and *FAM206A* as *ABITRAM.*

On the contrary, there were 42 DEGs among three treatment groups (the analysis *excludes the single patient who underwent combination therapy*): Immunotherapy [IT], Targeted Therapy [TT] and No Treatment [NT] (p < 0,0001, q < 0.05) (Fig. 1A). Of these, 37 genes were upregulated in the TT group and 5 genes were upregulated in the IT group. Interestingly, genes involved in the neurotrophic tyrosine kinase (NTRK) and epidermal growth factor receptor (EFGR) pathways, *NTRK3* and *amphiregulin* (*AREG)*, respectively, were increased in patients who relapsed subsequent to targeted therapy (Supplementary Table 1). Indeed, resistance to BRAF inhibitors can be caused by EGFR signalling [48,49], which justifies the combination of Vemurafenib with EGFR therapeutic inhibition (for example with Erlotinib). *AREG* encodes amphiregulin, a ligand for EGFR, which plays a role in mammary gland development [50]. AREG has long been known as an oncogenic driver and promotes self-sufficiency in growth signals, tissue invasion and inhibition of apoptosis, all of which promote tumour progression [51]. Previously, ‘driver-negative’ (i.e. the absence of BRAF, NRAS, KIT, GNAQ or GNA11 mutations) melanoma cell-lines that were less sensitive to trametinib and which displayed paradoxical activation of MEK1/2, were found to show basal EGFR activation due to AREG [52]. Targeting of AREG has been considered for other cancers, for example *AREG* siRNAs reduced EGFR activation in hepatocellular carcinoma cell lines and lung cancer cells, which led to decreased growth and increased sensitivity to anti-EGFR targeted therapy [53,54]. Targeting AREG could be beneficial in patients who relapse in response to targeted therapy.

It is of further interest to assess activation of the TRK pathway in melanoma in particularly in response to targeted therapy, where TRK activity increased in response to Vemurafenib therapy. Although fusion mutations of NTRK are known drivers of tumorigenesis, they are not invariably detected in melanoma but have been reported to be present in different subtypes of melanomas: 21–28.5% of spitzoid melanomas (a subtype of cutaneous melanoma with histopathological features of Spitz naevus (a benign skin tumour) [55] and to a lesser extent in acral (2.5%) (melanoma with predilection for acral areas, i.e. affecting peripheral areas of the body, mainly soles, palms, toes and fingers) [56] and cutaneous melanomas (~1%) (the major category of melanoma cases arising from sun exposure) [38,57–59]. It has been reported, that NTRK fusions and typical oncogenic drivers, such as BRAF, NRAS, GNAQ and GNA11 are mutually exclusive and NTRK fusions might be more common in BRAF or NRAS wild-type melanomas [60–62]. The increase of activity of the NTRK pathway here in response to BRAF inhibition is a novel observation and suggests that it might be beneficial to use Vemurafenib in combination with TRK inhibitors, larotrectinib or entrectinib.

67 DEGs were identified as significantly different between survival groups (Supplementary Table 2). Of these, 42 were upregulated and 25 were down-regulated in patients surviving <2 years (Fig. 1B). Reactome terms could be assigned to 28 of the 67 DEGs to 310 pathways, of which five reached the designated level of statistical significance (false discovery rate [FDR] <0.05) (Supplementary Table 3). These pathways included oncogene and oxidative stress induced senescence and four of these pathways were associated with a sole common gene, *CDKN2A. CDKN2A* gene was upregulated in the poor survival (< 2 years) cohort, which was somewhat surprising as loss of CDKN2A expression occurs frequently in primary invasive melanoma [63]. CDKN2A is often mutated in familial melanoma and is one of the major melanoma susceptibility genes, with its mutation allowing cells to escape from cell cycle arrest [64].

### RNA editing analysis

We further analysed the 67 RNAseq datasets to evaluate whether RNA editing events could show distinct clustering based on mutation status, patient survival or in response to targeted- or immunotherapy.

**Figure 2.**
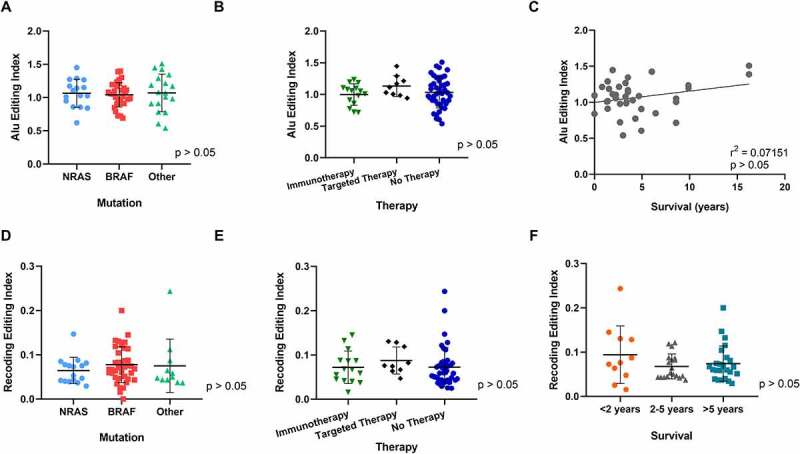
Comparison of A-to-I editing index and recoding editing index among 3 clinical groups: mutation, treatment and survival. (**A**) Alu editing index of 66 patients grouped into three mutation groups: BRAF, NRAS and other (which included WT (4), cKIT (4), GNAQ (1) and GNA11 (1). *Note one patient sample was excluded from analysis as it harboured a double mutation*. (**B**) Alu editing index of 66 Patients grouped into three therapy groups: after immunotherapy, after targeted therapy or prior to any therapy ‘No therapy’. *Note one sample with both immunotherapy and targeted therapy treatment prior to biopsy was excluded*. (**C**) Pearson correlation coefficient of editing versus survival in 37 patients. *Note living patients (n = 30) were excluded from analysis*. (**D**–**F**) Differences in the Recoding Editing Index (REI), defined as the weighted average over all known recoding sites from the REDIportal database, were analysed in the three clinical groups: mutation status (**D**), therapy response (**E**) and survival (**F**). One-way ANOVA analysis and Pearson correlation showed no significant differences in AEI among the clinical groups. One-way ANOVA analysis showed no significant differences in the REI in all groups tested.

In particular, we measured the global A-to-I RNA editing activity through the Alu editing index (AEI) metric, defined as the weighted average of editing events occurring in all adenosines within Alu elements and calculated using the RNAEditingIndexer tool [65]. A detailed analysis of AEI in cell lines organized in groups according to mutation status, therapy or survival revealed no significant differences in global Alu editing (Fig. 2A–C) and, thus, in the global editome. Additionally, we investigated potential differences in the Recoding Editing Index (REI), defined as the weighted average over all known recoding sites from the REDIportal database [66]. Although the REI index has been found deregulated in several tumours, for example in glioblastoma [67], our results showed no significant differences in melanoma based on mutation status, therapy or survival (Fig. 2D–F).

It was reported that in some cancers the loss of ADAR1 sensitizes tumours to immunotherapy [68]. Although expression levels in patients relapsed after immunotherapy and targeted therapy showed no significant up- or downregulation of ADAR1 in comparison to no treatment. As expected, however, RNA editing indexes showed correlation with the expression levels of genes coding ADAR enzymes (Supplementary Figure 2A, B). The AEI showed significant positive correlation with *ADAR1* gene expression (r(65) = 0.300, p = 0.014), whereas the REI index correlated positively with *ADARB1* gene (coding ADAR2) expression (r(65) = 0.329, p = 0.0065).

While the vast majority of editing events take place in repeated regions and most specifically in Alu rich segments [69], several editing events occur in coding regions of RNAs, with some of them leading to amino acid substitutions (recoding events) [4]. Thus, in addition to global editing indexes detailed above, we studied editing at individual sites. In order to investigate relevant sites in different RNA regions, we separated A-to-I editing sites into two categories; whether they were located in non-repetitive regions (including coding regions) or residing in Alu repetitive elements. We then focused only on editing events showing levels higher than 30%. In this way, we filtered 8,005 edited sites in non-repetitive regions (recoding editing) and 65,513 edited sites in Alu regions. Editing frequency comparison was performed in each of these locations and in the aforementioned three clinical groups: mutation status, survival or therapy response.

**Figure 3.**
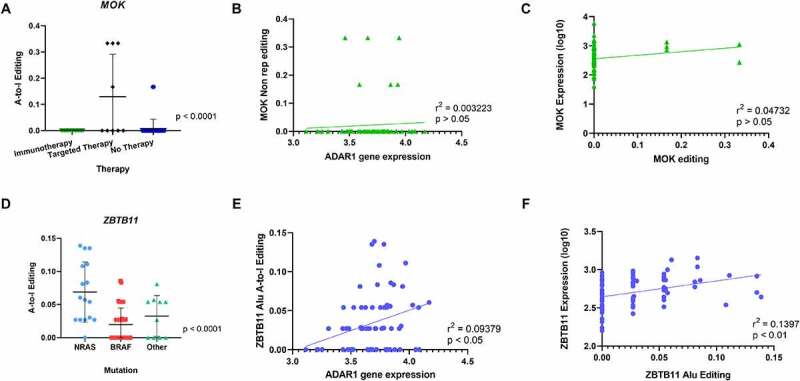
Comparison of A-to-I editing among 3 clinical groups: mutation, treatment and survival. Editing frequency comparison was performed in non-repetitive regions and Alu repetitive elements in the three clinical groups: mutation status, therapy response and survival. (**A**) One-way ANOVA analysis showed significant differences in A-to-I Editing of the *MOK* gene in non-repetitive regions in tumours from patients who relapsed during targeted therapy. (**B**) Pearson Correlation of *MOK* editing with ADAR1 gene expression showed no statistical significance. (**C**) No correlation of MOK A-to-I editing in non-repetitive regions with gene expression. (**D**) One-way ANOVA analysis showed significant differences in A-to-I Editing of the *ZBTB11* gene in Alu regions in tumours from patients in the NRAS mutation group. (**E**) Positive Correlation of ZBTB11 A-to-I editing with ADAR1 gene expression. (**F**) Positive correlation of ZBTB11 A-to-I editing with *ZBTB11* gene expression.

The analysis of recoding editing revealed that one gene was significantly hyper-edited from A-to-I in the Therapy group. Indeed, the *MAPK/MAK/MRK overlapping kinase (MOK*) mRNA was significantly more edited in tumours from patients who relapsed during targeted therapy (Fig. 3A). However, editing of this gene did not correlate neither with gene expression levels of ADAR1 nor with its own gene expression (Fig. 3B) and C. MAP kinases are critical in signal transduction pathways involved in the regulation of cellular proliferation, differentiation and apoptosis [70]. The MOK protein is encoded by the receptor for advanced glycation endproducts1 (*RAGE1)* gene, which has been considered as a tumour-associated antigen for its wide expression in various tumours, including melanoma [71], renal cell carcinoma [72], head and neck cancer [73], mesothelioma [74], hepatocellular carcinoma [75] and acute myeloid leukaemia [76], but silenced in normal tissues with the exception of the retina [71]. Moreover, MOK belongs to the same MAPK superfamily, as MEK1 and MEK2 proteins [70,77], which have crucial roles in tumorigenesis and whose inhibition is currently an attractive strategy in melanomas with BRAF mutation [78]. In clinical practice, the majority of patients are intrinsically resistant or rapidly acquire resistance to MAPK pathway inhibition and immune checkpoint blockade treatment [79–81]. The lack of response is interpreted by new mutations and non-mutational events in tumour cells [81]. Our observations suggest that in the context of targeted therapy treatment with MEK inhibitors, hyper-editing of *MOK* could be a possible marker for treatment-resistance.

In further analyses of recoding editing, both mutation type (Supplementary Figure 3A) and survival rate (Supplementary Figure 3B) showed no statistically significant editing pattern.

When focusing on editing in repetitive Alu regions, two genes showed hyperediting in Mutation and Therapy groups. Firstly, *zinc finger and BTB domain containing 11* (*ZBTB11*) was identified as being hyper-edited in the NRAS mutation group, when compared to melanoma samples with BRAF or other mutations (Fig. 3D). Editing levels of *ZBTB11* showed some slight positive correlation with ADAR1 gene expression levels, suggesting the ADAR1 editing activity in this gene (Fig. 3E). Interestingly, gene expression of ZBTB11 correlated with RNA editing (Fig. 3F). ZBTB11 is a member of a family that consists of approximately 49 ZBTB proteins [82]. Some ZBTB members can function as vital proto-oncogenes such as ZBTZ27, ZBTB71 [83,84], while others exert tumour suppressive roles, including ZBTB29, ZBTB28 and ZBTB16 [83,85,86]. Interestingly, an inverse relationship between some of these genes was shown, where ZBTB27 loses its cancer promoting function when ZBTB28 expression is present [86]. A downregulation of ZBTB11 was also reported in hepatocellular carcinoma samples [87]. ZBTB11 (and ZFP131) preserves pluripotency of embryonic stem cells by pausing RNA Polymerase II at pro-differentiation genes [88]. This is interesting considering the role of the NRAS-MAPK pathway in maintaining pluripotency in stem cells [89]. Further studies could highlight whether hyper-editing is a consequence or an accompanying event in mutagenesis by mutations in the NRAS-MAPK pathway.

**Figure 4.**
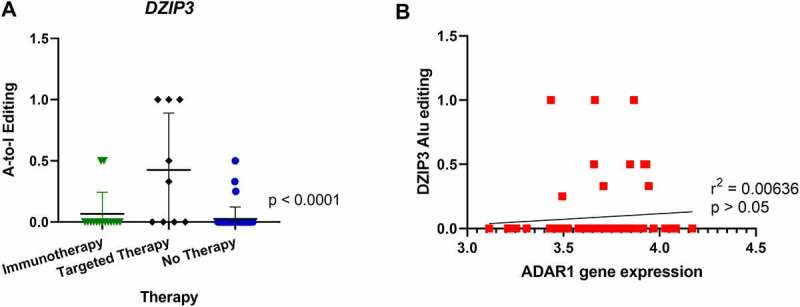
Comparison of A-to-I editing of DZIP3 and correlation with ADAR1 expression. (**A**) One-way ANOVA analysis showed significant differences in A-to-I editing of the *DZIP3* gene in Alu regions in tumours from patients relapsing after targeted therapy. (**B**) Pearson correlation of *DZIP3* Alu editing with ADAR1 gene expression showed no statistical significance.

Additionally, editing in the transcript encoding *deleted in azoospermia (DAZ)-interacting protein 3* (*DZIP3*) was also significantly increased in patients who relapsed after treatment with targeted therapy prior to sampling (Fig. 4A). No correlation was found when analysing *DZIP3* editing and ADAR enzyme expression levels or *DZIP3* editing and *DZIP3* expression levels (Fig. 4B). In further analysis of A-to-I editing no further significant differences were found in editing patterns when comparing survival of patients (Supplementary Figure 3C). *DZIP3* is known to bind to the coactivator-associated arginine methyltransferase 1 (CARM1) protein, which promotes oestrogen receptor α-mediated transcription in breast cancer [90]. Interestingly, *DZIP3* has been shown to stabilize Cyclin D1 (CCND1) expression, which promotes cell-cycle progression and proliferation of cancer cells [91]. CCND1 amplifications in melanoma are associated with resistance to checkpoint inhibitors [92]. To our knowledge, no association of *DZIP3* with melanoma has been reported to date.

Previously, we identified Alu hyper-editing of Gap junction gamma-1 (GJC1) protein in relapsed tumour cell-lines after immunotherapy [45]. Though GJC1 is not evident in our dataset here, this might be due to the smaller sample size we used previously, or evolution of the DARNED database and analysis tools and a higher editing threshold employed in the current study.

Mutations in DNA have been long known to be fundamental in the initiation and progression of cancers [93]. RNA modifications by RNA editing of tumour-associated genes or by changes of RNA editing levels are also widespread in cancer genomes and may contribute to cancer pathogenesis [1–4]. In this study, we aimed to assess differential gene expression and RNA editing events in melanoma associated with mutation status, treatment and survival. Collectively, our findings provide insights into RNA editing events in human malignant melanoma as assessed by measuring the A-to-I editing status of its specific targets in relation to clinical outcomes. Our findings suggest that editing events in melanoma might have clinical utility in the identification of cancer biomarkers, which could be further validated in future studies. Additionally, the role of ZBTB11, MOK and DZIP3 in melanocytic tumours requires further clarification. Whether therapeutically targeting the ZBTB11 pathway would have a favourable outcome in NRAS mutated melanoma would require experimental evaluation. In order to optimize current treatment possibilities and lower the risks of relapse during treatment it would be important to collect more evidence in editing patterns of melanoma, specifically taking into account its relation to patients’ response to therapy.

Our present work sheds new light on both gene expression and RNA editing in the context of relapse during cancer therapies and might be the basis for new diagnostic and therapeutic approaches.

## Methods

### Primary human metastatic melanoma tissue samples

This study was conducted in approval of Kantonale Ethikkomission under approval number KEK‐ZH Nr. 2014‐0425, EK647 and EK800. Informed consent for investigational use was obtained prior to biopsy collection. To compare RNA editing between melanoma tumour tissues we collected data from 67 patients diagnosed with stage III–IV metastatic melanoma. Biopsies were collected following surgical resection as a treatment to relapsing melanoma at the University Hospital of Zürich. In order to generate melanoma cell lines from native material, we applied the workflow as previously described by Raaijmakers *et al* [46]. Briefly, cell lines were subjected to a ‘no splitting, limited cell culture change’ approach, where medium was changed only once every 2 weeks. Furthermore, a ‘short trypsinization and selective adherence’ method was used to detach melanoma cells and avoid detachment of fibroblasts. Oncogenic mutation status and morphology was confirmed to determine that the original tumour heterogeneity was retained [46]. All research on human material were conducted in accordance with the Declaration of Helsinki and Swiss law.

### RNA extraction and sequencing

High-quality DNA was used to prepare a customized target library using the Nimblegen SeqCap EZ kit (Nimblegen). Ilumina Hiseq 4000 was used to perform sequencing of 0,125 lanes per sample, paired end, with 150 bp reads [46]. Cell line purity was estimated based on the Mutant allele frequency (MAF) calculated as follows: MAF = mutant copies/(wild-type copies + mutant copies). Mutations in BRAF (V600E) and NRAS (Q61R, Q61K, Q61L) in melanoma are reported to be heterozygous, and thus cell line purity was calculated as 2*MAF. High-quality RNA was extracted with QIAGEN RNeasy kit. RNA capture was performed with TruSeq RNA library Prep Kit v2 (Illumina). RNA sequencing was sequenced at 100 bp single ends on a HiSeq4000 at the FGCZ.

### Statistical analyses

Datasets of 67 samples were available for analysis. Raw reads were trimmed by fastp (version 0.20.0) [94] in order to remove adapters and low-quality regions. Cleaned reads were then aligned onto the human reference genome (assembly GRCh-38) using the ultrafast STAR program (version 2.5.2b) [95]. Gene quantification was carried out using Salmon (version 0.14.1) [96]. A differential gene expression analysis was performed using Qlucore software v 3.6. with R script for limma-voom [97]. RNA editing per sample was profiled using REDItools and more than 4.5 millions of known events from the REDIportal database [98]. A-to-I events were selected according to the following criteria: qPhred score (base quality score) ≥25, mapping quality score ≥20 [99]. The obtained RNA editing results were then analysed with Qlucore Omics Explorer v.3.6 (www.qlucore.com) focusing on editing events with levels ≥30%. Recoding editing was analysed separately with the exclusion of known SNPs from dbSNP. Principal component analysis plots and heat maps were generated with Qlucore software. Editing events were analysed with applying unpaired t-test for two groups and ANOVA test for multiple group comparison. For each comparison P < 0.01, q < 0.05 was considered statistically significant, with at least three independent experiments performed for each analysis. The Pearson Correlation coefficient was calculated using GraphPad Prism 8.4.0, with p < 0.05 considered as significant. AEI index was calculated using the RNAEditingIndexer while REI index was quantified using a python script as described in [100].

## Supplementary Material

Supplemental Material

## Data Availability

The datasets analysed during the study will be uploaded to GEO.

## Abbreviations

ADAR, adenosine deaminase acting on RNA; ADAT, adenosine deaminase acting on tRNA; AID/APOBEC, cytidine deaminase/apolipoprotein B editing complex; Alu, Arthrobacter luteus; AREG, Amphiregulin; ARF, Alternative Reading Frame; APC, Adenomatous polyposis coli; AZIN1, Encoding antizyme inhibitor 1; CARM1, Coactivator-associated arginine methyltransferase 1; cKIT, Receptor tyrosine kinase; CDC14B, Cell division cycle 14B; CDK4/6, Cyclin dependent kinases 4 and 6; CDKN2, Cyclin-dependent kinase inhibitor2A; COG3, Component of oligomeric Golgi complex 3; COPA, Coatomer protein complex subunit α; CTLA-4, Cytotoxic T-lymphocyte-associated protein 4; CTNNB1, Catenin Beta 1; DZIP, Deleted in azoospermia (DAZ)-interacting protein 3; EGFR, epidermal growth factor receptor; GABRA3, gamma-aminobutyric acid type A receptor alpha 3 subunit; GJC1, Gap junction gamma-1; Glur-B, glutamate receptor subunit B; GNA11, G Protein Subunit Alpha 11; GNAQ, G Protein Subunit Alpha Q; IGFBP7, Insulin-like growth factor-binding protein 7; MAPK, Mitogen-activated protein kinase; MEK, Mitogen-activated protein kinase kinase; MEKi, MEK inhibitor; NEIL1, Nei like DNA glycosylase 1; NTRK, Neurotrophic Tyrosine Kinase; PD-1, Programmed cell death-1 receptor; PD-L1, Programmed cell death-ligand 1; PODXL, Podocalyxin like; RAGE1, Receptor for advanced glycation endproducts 1; RHOQ, Ras homologue family member Q; RIG-I, Retinoic acid-inducible gene I; SINEs, Short interspersed nuclear elements; SLC22A3, Solute carrier family 22 member 3; ZBTB11, Zinc finger and BTB domain containing 11
