# Supplementary material for: The A to I editing landscape in melanoma and its relation to clinical outcome
Source: RNA Biol. 2022 Aug 21;19(1):996–1006. doi: 10.1080/15476286.2022.2110390 (PMC9415457; doi:10.1080/15476286.2022.2110390)
Supplement: Supplemental Material [file KRNB_A_2110390_SM4097.zip › Supp Table 1.docx]

**Supplementary Table 1. Table of 42 statistically significant DEGs between treatment groups.** 37 DEGs were significantly increased in patients who relapsed after targeted therapy (highlighted in bold).

| **Gene** | **P-value** | **FDR** | **Gene** | **P-value** | **FDR** | **Gene** | **P-value** | **FDR** | |
| --- | --- | --- | --- | --- | --- | --- | --- | --- | --- |
| **ADRA1D** | 3.908e-06 | 0.008 | **FOXF1** | 6.897e-05 | 0.042 | RP11-575F12.3 | 6.243e-05 | 0.040 |  |
| **AMIGO2** | 3.721e-05 | 0.030 | **GABRA2** | 6.253e-05 | 0.040 | RP11-752G15.4 | 2.372e-05 | 0.024 |  |
| **AREG** | 1.495e-05 | 0.019 | **GALNT13** | 7.047e-06 | 0.013 | **SLAMF8** | 1.910e-06 | 0.006 |  |
| **BTBD11** | 1.901e-05 | 0.020 | **GALNT6** | 1.784e-05 | 0.020 | **SLC15A3** | 5.879e-05 | 0.040 |  |
| C5orf63 | 5.661e-05 | 0.040 | **HS3ST3B1** | 3.300e-05 | 0.030 | **SOX11** | 3.726e-05 | 0.030 |  |
| **CACNG8** | 1.313e-07 | 0.001 | **IGFBPL1** | 7.843e-05 | 0.045 | **SPOCK3** | 1.156e-05 | 0.017 |  |
| **CD1D** | 3.869e-05 | 0.030 | **IL18R1** | 2.229e-07 | 0.001 | **SVEP1** | 1.804e-05 | 0.020 |  |
| **COL4A5** | 1.463e-05 | 0.019 | **MID2** | 6.185e-05 | 0.040 | **TMEM37** | 7.672e-08 | 0.001 |  |
| **DOK6** | 1.805e-06 | 0.007 | **NOVA1** | 4.429e-09 | 0.0001 | **TOR4A** | 6.860e-05 | 0.0418 |  |
| **EFNB1** | 6.223e-05 | 0.040 | **NTRK3** | 3.371e-05 | 0.030 | TRA2A | 3.534e-05 | 0.030 |  |
| **ELAVL2** | 4.857e-07 | 0.002 | **PCDHB17P** | 3.571e-06 | 0.008 | **TREM1** | 1.063e-05 | 0.016 |  |
| **FAM84A** | 3.676e-06 | 0.008 | **PI3** | 3.030e-05 | 0.030 | **TRIM54** | 8.218e-06 | 0.0139 |  |
| **FCAMR** | 7.148e-05 | 0.042 | POU6F2 | 4.298e-06 | 0.008 | **TSPAN18** | 1.191e-05 | 0.017 |  |
| **FER1L4** | 3.861e-05 | 0.030 | **RP11-191A15.2** | 2.507e-06 | 0.007 | **ZNF582** | 8.709e-05 | 0.050 |  |
