# Supplementary material for: The A to I editing landscape in melanoma and its relation to clinical outcome
Source: RNA Biol. 2022 Aug 21;19(1):996–1006. doi: 10.1080/15476286.2022.2110390 (PMC9415457; doi:10.1080/15476286.2022.2110390)
Supplement: Supplemental Material [file KRNB_A_2110390_SM4097.zip › Supp Table 2.docx]

**Supplementary Table 2. Table of 67 statistically significant DEGs between survival groups.** Of these, 42 were upregulated (highlighted in bold) and 25 were down-regulated in patients surviving <2 years.

| **Gene** | **P-value** | **FDR** | **Gene** | **P-value** | **FDR** | **Gene** | **P-value** | **FDR** |
| --- | --- | --- | --- | --- | --- | --- | --- | --- |
| AADACL2 | 3.163e-05 | 0.030 | **HRSP12** | 6.554e-05 | 0.036 | PEG10 | 3.088e-05 | 0.030 |
| AC015971.2 | 8.712e-05 | 0.042 | **HSD17B10** | 2.580e-05 | 0.030 | **PGM3** | 0.0001 | 0.045 |
| **AC093642.3** | 1.963e-06 | 0.012 | HSD17B7P2 | 4.313e-05 | 0.030 | **PKIB** | 4.358e-05 | 0.030 |
| **AC104653.1** | 6.373e-05 | 0.036 | IQCC | 0.0001 | 0.050 | **PKP3** | 2.872e-05 | 0.030 |
| AC104777.2 | 1.871e-05 | 0.030 | **ISCA1** | 3.206e-05 | 0.030 | **PSMB1** | 3.852e-05 | 0.030 |
| **AMZ1** | 9.736e-05 | 0.042 | **ITK** | 4.393e-05 | 0.030 | **RNF113A** | 0.0001 | 0.050 |
| **ARMC6** | 5.903e-06 | 0.020 | KB-1615E4.2 | 2.641e-05 | 0.030 | **RP11-130F10.1** | 2.469e-05 | 0.030 |
| ARPIN | 6.237e-05 | 0.036 | **LINC01060** | 1.923e-06 | 0.012 | RP11-199F11.2 | 6.645e-05 | 0.036 |
| **ASS1P1** | 1.936e-06 | 0.012 | **LINC01237** | 9.968e-05 | 0.042 | RP11-343B22.2 | 1.222e-05 | 0.024 |
| BTBD3 | 4.943e-05 | 0.032 | **LRRC8E** | 4.236e-06 | 0.017 | **RP11-382M14.1** | 2.230e-05 | 0.030 |
| **C19orf43** | 8.189e-05 | 0.041 | **MCEE** | 8.136e-05 | 0.0414 | RP11-631M6.2 | 2.396e-05 | 0.030 |
| **C19orf53** | 0.0001 | 0.049 | **MED30** | 4.565e-05 | 0.030 | RP11-764K9.4 | 9.115e-05 | 0.042 |
| **C8orf59** | 0.0001 | 0.049 | **MICALCL** | 5.561e-05 | 0.035 | **RPL35** | 8.818e-05 | 0.042 |
| **C9orf85** | 4.165e-05 | 0.030 | **NANS** | 6.277e-05 | 0.036 | **TMEM141** | 3.367e-05 | 0.030 |
| **CDKN2A** | 7.927e-06 | 0.024 | NCOR1 | 2.495e-05 | 0.030 | **TTC13** | 1.546e-07 | 0.004 |
| **CLDN14** | 1.215e-05 | 0.024 | **NDUFB6** | 3.414e-05 | 0.0230 | TTC19 | 4.103e-05 | 0.030 |
| CTC-523E23.3 | 0.0001 | 0.045 | **NPIPB15** | 0.0001 | 0.045 | **UXT** | 8.721e-05 | 0.042 |
| CTD-2145A24.5 | 9.278e-05 | 0.042 | **NQO2** | 4.203e-05 | 0.030 | ZBTB39 | 0.0001 | 0.045 |
| CTD-2210P24.3 | 1.039e-05 | 0.024 | **NSMCE1** | 9.881e-05 | 0.042 | ZFPM2 | 1.221e-05 | 0.024 |
| DENND5B | 9.417e-05 | 0.042 | **OPLAH** | 5.721e-05 | 0.035 | ZNF112 | 7.378e-05 | 0.039 |
| EWSAT1 | 0.0001 | 0.049 | **ORC3** | 4.027e-05 | 0.030 | ZSWIM7 | 4.052e-05 | 0.030 |
| **FAM104B** | 0.0001 | 0.049 | OSBPL7 | 9.887e-05 | 0.042 |  |  |  |
| **FAM206A** | 3.783e-06 | 0.017 | **PCLO** | 3.387e-05 | 0.030 |  |  |  |
