# Supplementary material for: The A to I editing landscape in melanoma and its relation to clinical outcome
Source: RNA Biol. 2022 Aug 21;19(1):996–1006. doi: 10.1080/15476286.2022.2110390 (PMC9415457; doi:10.1080/15476286.2022.2110390)
Supplement: Supplemental Material [file KRNB_A_2110390_SM4097.zip › Supp Table 3.docx]

**Supplementary Table 3. Reactome pathway analysis of 67 statistically significant DEGs between survival groups.** Reactome terms could be assigned to 28 of the 67 DEGs to 310 pathways, of which five reached the designated level of significance (false discovery rate [FDR], <0.05).

| **Pathway name** | **Entities in**  **reference list** | **Entities in**  **analyzed list** | **Genes** | **P-value** | **FDR** |
| --- | --- | --- | --- | --- | --- |
| Oncogene Induced Senescence | 42 | 5 | CDKN2A | 2.39e-06 | 0.001 |
| Transcriptional regulation by VENTX | 48 | 4 | CDKN2A | 1.02e-04 | 0.015 |
| Diseases of Cellular Senescence | 4 | 2 | CDKN2A | 1.91e-04 | 0.015 |
| Diseases of Cellular Response to Stress | 4 | 2 | CDKN2A | 2.75e-04 | 1.91e-04 |
| Oxidative Stress Induced Senescence | 114 | 5 | CDKN2A | 2.68e-04 | 0.017 |
