# Supplementary figures and images for: The A to I editing landscape in melanoma and its relation to clinical outcome
Source: RNA Biol. 2022 Aug 21;19(1):996–1006. doi: 10.1080/15476286.2022.2110390 (PMC9415457; doi:10.1080/15476286.2022.2110390)

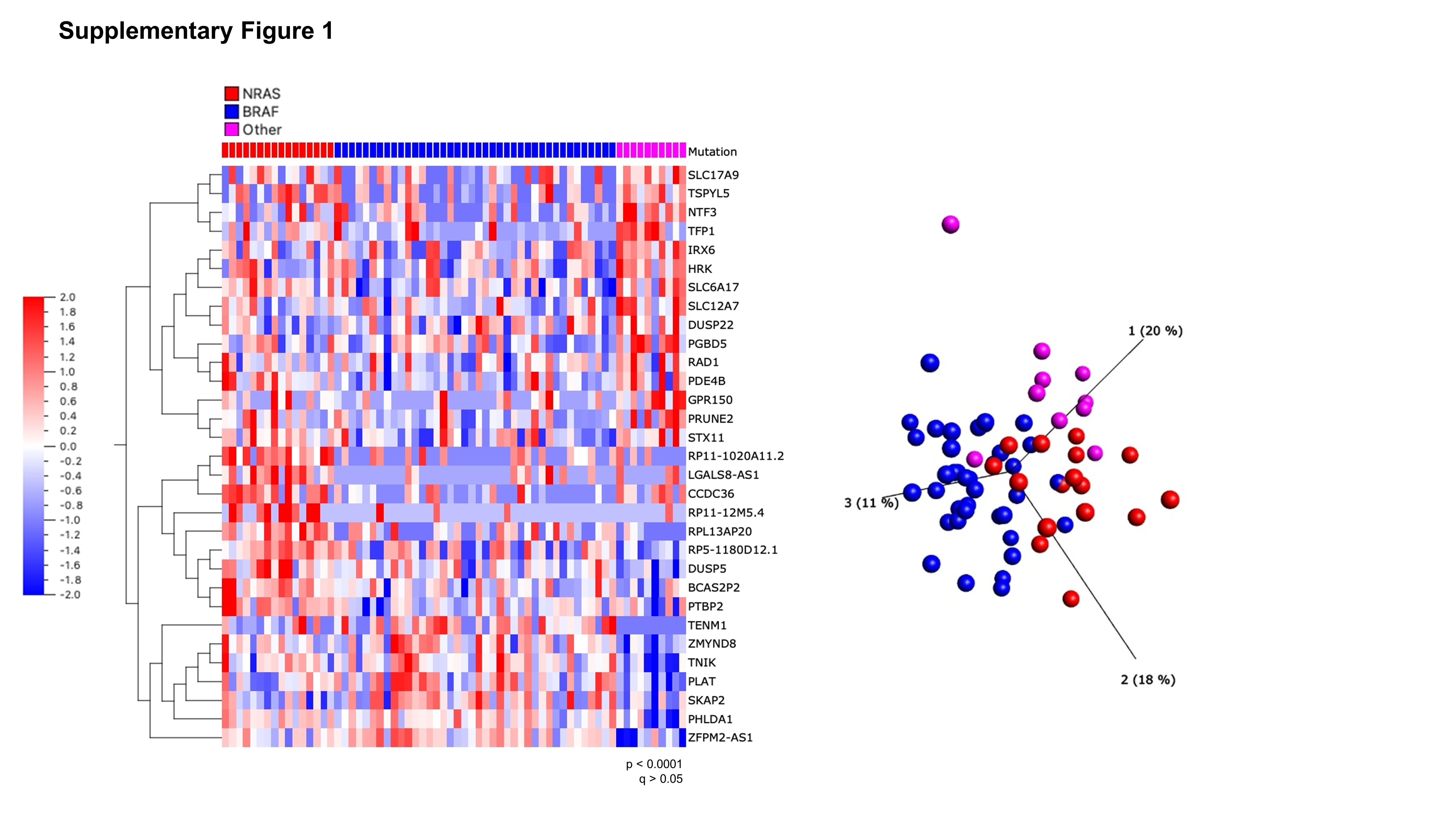

Supplement: Supplemental Material [file KRNB_A_2110390_SM4097.zip › Supp Fig 1.tif]

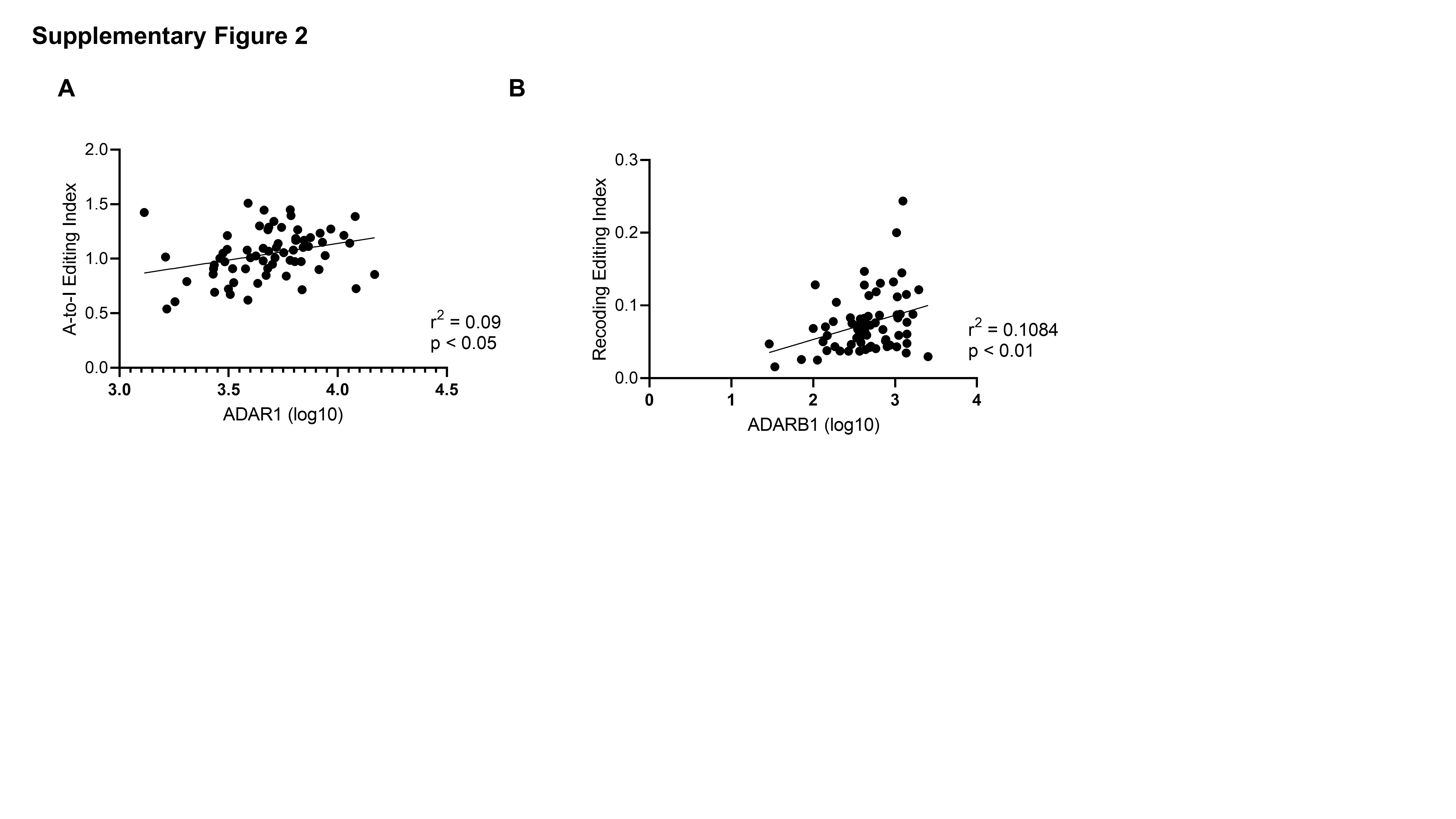

Supplement: Supplemental Material [file KRNB_A_2110390_SM4097.zip › Supp Fig2.tif]

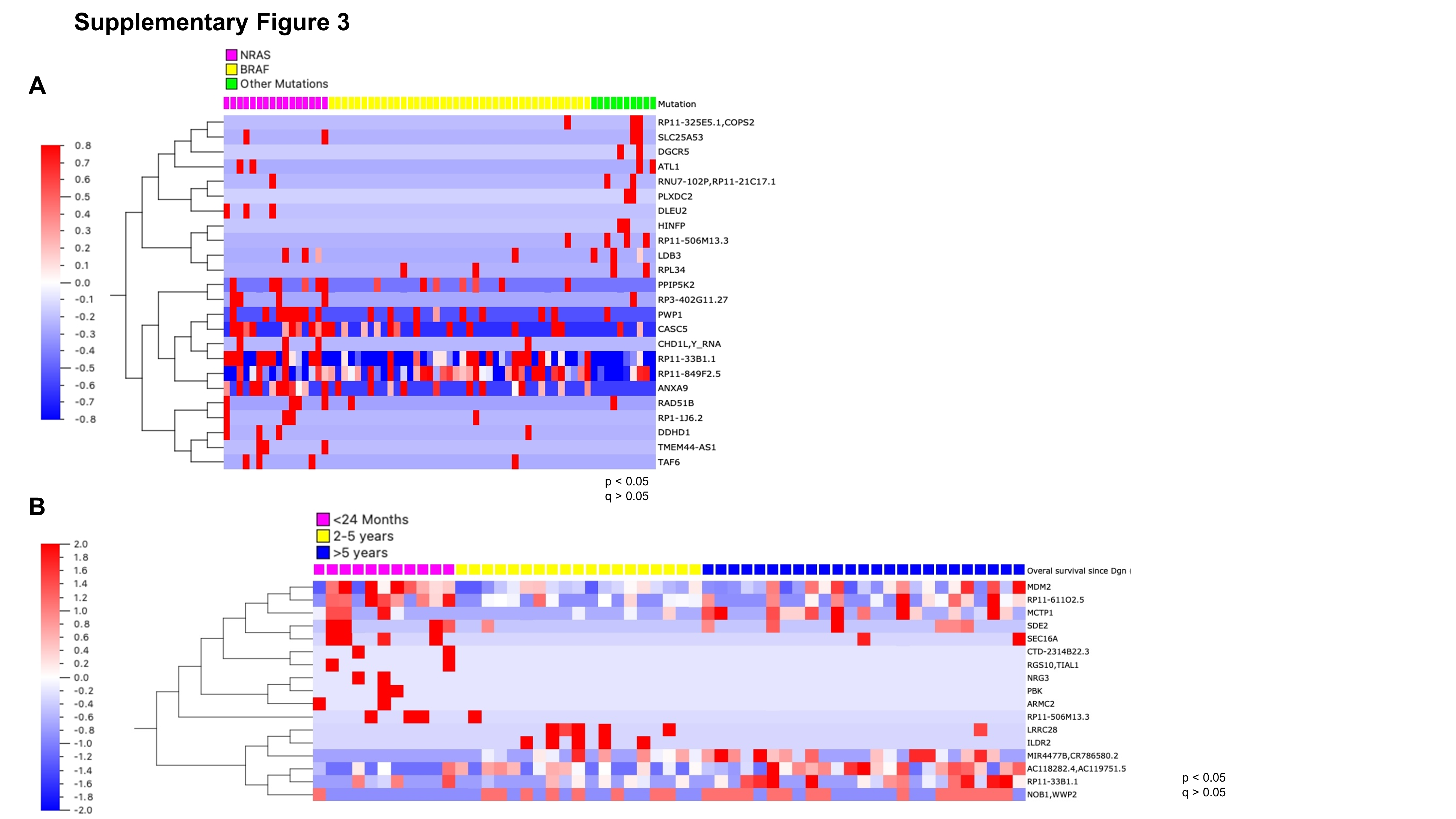

Supplement: Supplemental Material [file KRNB_A_2110390_SM4097.zip › Supp Fig3 A and B.tif]

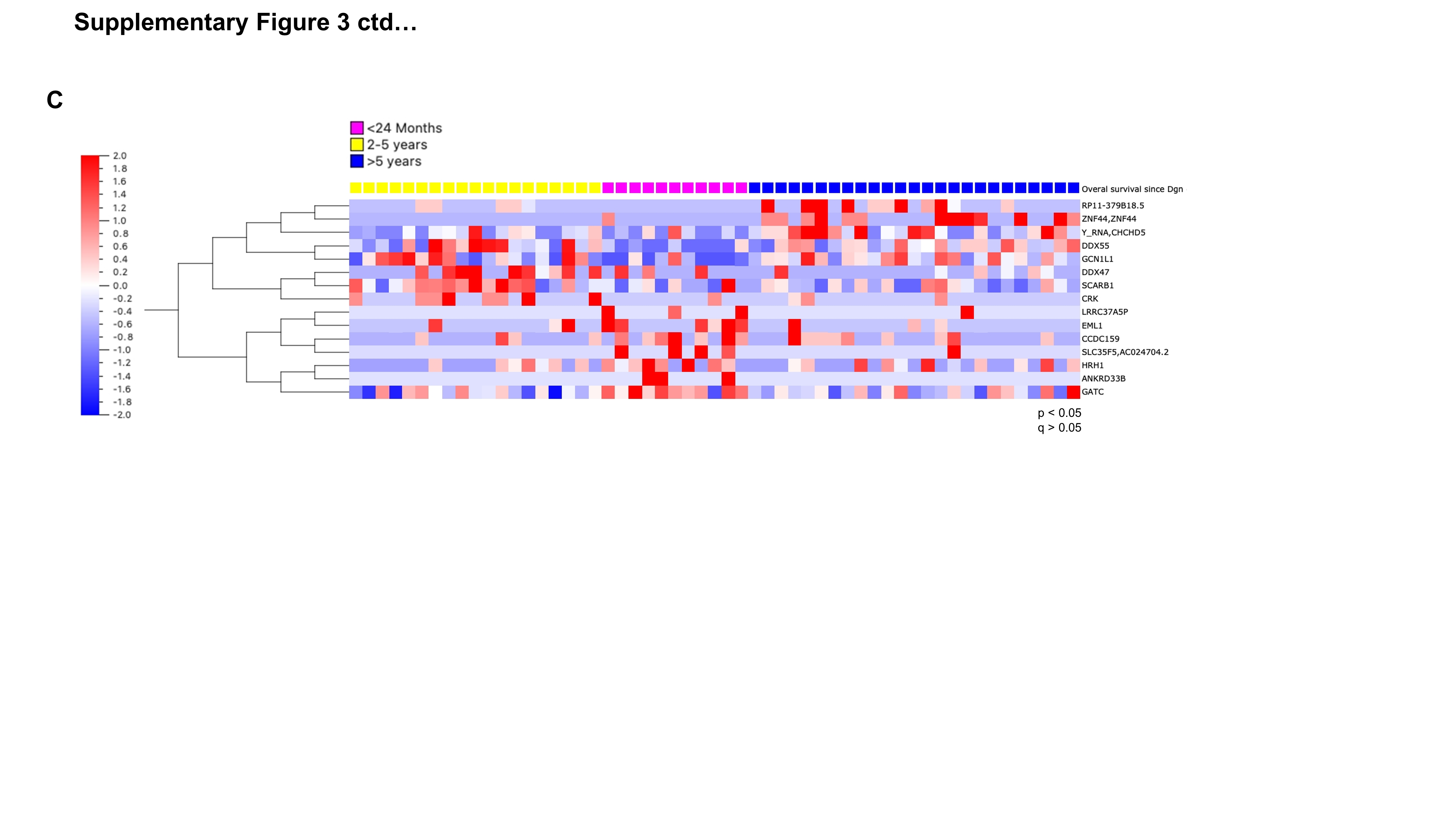

Supplement: Supplemental Material [file KRNB_A_2110390_SM4097.zip › Supp Fig3 C.tif]
